# La syphilis secondaire: la grande simulatrice

**DOI:** 10.11604/pamj.2013.15.52.2676

**Published:** 2013-06-11

**Authors:** Naoufal Hjira, Mohammed Boui

**Affiliations:** 1 Service de dermatologie, hôpital militaire d'instruction Mohammed V, Rabat, Maroc

**Keywords:** Syphilis secondaire, infection sexuellement transmissible, treponema palidum, papules érythémateuses, secondary syphilis, sexually transmitted infection, treponema palidum, erythematous papules

## Image en médecine

La syphilis est une infection sexuellement transmissible due au *Treponema palidum*. Elle est en forte recrudescence en rapport avec la pandémie du VIH; caractérisée par son polymorphisme clinique, elle est appelée la grande simulatrice. La bénignité des formes précoces et la gravité des formes tardives soulignent l'intérêt du diagnostic et du traitement précoce. Son diagnostic est sérologique, son traitement est facile, efficace et peu onéreux, mais le meilleur traitement est la prévention. Nous rapportons le cas d'un patient âgé de 52 ans, marié et père d'un enfant, il n'a pas d'antécédents pathologiques particuliers, il consulte pour des lésions du cuir chevelu apparues il y'a deux mois à type de papules érythémateuses, découvertes après une sensation de prurit et après avoir rasé le crâne. L'examen clinique trouve des papules érythémateuses cuivrées, excoriées et infiltrées, occupant le vertex et les régions temporales, sur le reste du tégument nous découvrons des lésions similaires sur le scrotum et sur les régions palmo-plantaires, sans atteinte muqueuse, la sérologie syphilitique était positive: TPHA 1/1280 VDRL 1/64. L'aspect clinique des lésions évoquait un eczéma une dermite séborrhéique ou un prurigo, mais la positivité de la sérologie syphilitique et la disparition totale des lésions sous extencilline 1.2 M en deux injections simultanées et répétées après une semaine, nous a permis de retenir le diagnostic de syphilis secondaire. Les sérologies des hépatites B, C et du VIH sont négatives chez notre patient. Les sérologies réalisées chez l'épouse sont négatives.

**Figure 1:**
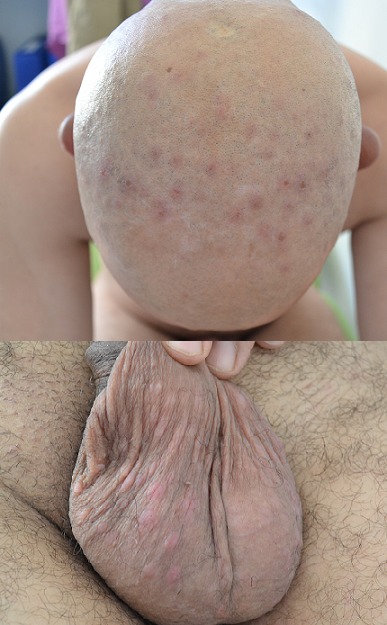
Papules érythémateuses excoriées du cuir chevelu et papules érythémateuses infiltrées scrotales

